# A Framework to Implement IoT Network Performance Modelling Techniques for Network Solution Selection †

**DOI:** 10.3390/s16122038

**Published:** 2016-12-01

**Authors:** Declan T. Delaney, Gregory M. P. O’Hare

**Affiliations:** 1 School of Computer Science, University College Dublin, Belfield, Dublin 4, Ireland; gregory.ohare@ucd.ie; 2 Earth Institute, University College Dublin, Belfield, Dublin 4, Ireland

**Keywords:** Internet of Things, performance, modelling, standardised testing, framework

## Abstract

No single network solution for Internet of Things (IoT) networks can provide the required level of Quality of Service (QoS) for all applications in all environments. This leads to an increasing number of solutions created to fit particular scenarios. Given the increasing number and complexity of solutions available, it becomes difficult for an application developer to choose the solution which is best suited for an application. This article introduces a framework which autonomously chooses the best solution for the application given the current deployed environment. The framework utilises a performance model to predict the expected performance of a particular solution in a given environment. The framework can then choose an apt solution for the application from a set of available solutions. This article presents the framework with a set of models built using data collected from simulation. The modelling technique can determine with up to 85% accuracy the solution which performs the best for a particular performance metric given a set of solutions. The article highlights the fractured and disjointed practice currently in place for examining and comparing communication solutions and aims to open a discussion on harmonising testing procedures so that different solutions can be directly compared and offers a framework to achieve this within IoT networks.

## 1. Introduction

The Internet of Things (IoT) paradigm is quickly maturing to a state were the technology is being adopted in industry for a wide range of applications including medical [1], precision agriculture [2] and Building Energy Management System (BEMS) [3]. An IoT solution allows for quick and increasingly cost effective deployment of sensors to gather data and perform actions within a controlled environment. An underlying wireless communications network facilitates the collection of data and delivery of commands to the necessary locations. This network is responsible for delivering reliable, efficient and timely communications within an inherently dynamic environment. Difficult communication conditions, often experienced in IoT networks, can lead to a trade off between delivering these requirements in the network. Not only does each new application demand a different type of trade off between requirements, but each different environment will also effect how a network can deliver the requirements. Obtaining the required response from the network for each application is essential to the adoption of IoT technologies and is regarded as one of the main challenges facing IoT networks [4]. This challenge is currently being addressed with many new communications solutions entering the IoT landscape. This, however, introduces a wide variety of different communications solutions many of which have been tested and evaluated under disparate environmental conditions. This presents two further issues for industry application development:

- Application developers will not have the network experience or expertise to choose the best solution for the given application or environment leading to reduced performance.
- It is difficult to directly compare solutions. Each prospective solution, therefore, must be tested within the specific deployment environment to ensure the best solution. This incurs additional time and cost per deployment.

This article presents a framework which can be used to address these issues presented for industry application development. The framework constitutes a testing procedure and system to autonomously choose a communications solution for a network. This creates a layer of abstraction on top of the network layer for the application developer. The application developer must only define the preferred requirements set for the application leaving the system to evaluate and choose the most appropriate solution for the deployment. The framework uses a modular design consisting of four main tenets:

- **Maintaining a solution repository:** Collate a set of data from a wide range of communications solutions each evaluated under the same conditions and criteria. This provides a consistent set of data to compare each solution.
- **Solution modelling:** Model the response of each of the communications solutions under the changing conditions for the purpose of prediction. The model can be used by the system to easily predict how a solution will perform in a given set of conditions and compare with competing solutions.
- **Analysing deployment:** As a prediction model is in place, only the environmental conditions of the deployment need be collected. The system can use simple and minimally invasive means to collect the current environmental conditions.
- **Solution selection and deployment:** Based on the current conditions, choose and deploy the most appropriate solution given the competition in predicted performance.

The implemented system is dependent on the ability of the chosen environmental variables to characterise the response effectively and the suitability of the modelling technique to model that response. While the main purpose of the article is to present the framework as a whole, the framework is demonstrated using a defined set of evaluation metrics, network condition variables used to characterise the response in the network and modelling tools used in prediction modelling. The article uses the framework to evaluate the suitability of using the physical features exhibited by the network as the condition variables that best characterise the response elicited by a communications solution in the network. If the physical features selected are suitable for this stated purpose, a set of solutions tested under a range of these physical features will give a rich and accurate response suitable for directing solution selection. Modelling is performed using linear regression modelling techniques on the test data. Using the framework we can begin to answer two important questions:

- Is it possible to effectively predict how well a communications solution will perform compared to another solution within an arbitrary deployment using the physical features as predictor variables.
- From such predictions is it possible to either advise on, or automatically deploy the most appropriate solution for an application.

The questions above are subject to how we define the term *well*, how we characterise an *arbitrary deployment* and how we determine *most appropriate*. For the purpose of this article we use four evaluation metrics: latency, reliability, efficiency and stability to determine how *well* a solution performs. An *arbitrary deployment* is a previously untested network which is characterised by a number of measurable physical attributes of the network based around the network size, node density, network diameter and link conditions. The *most appropriate* solution is chosen using a set of requirements, the same as those used for evaluation, which the application requests from the network. Even with a defined set of requirements it is difficult to determine what is *most appropriate* for the application. We begin with a strict but narrow definition for most appropriate; the best performing communications solution for each evaluation metric using the prediction model. Simply, from our set of prediction models can we generate a ranked list of the best performing solutions for each evaluation metric. This is then expanded to a more general discussion on selecting the solution based on how the whole response matches the application requirements. It is important to note that as a modular framework, each facet is interchangeable for use with another technique. For example, in this article linear regression modelling is chosen to model the data obtained from the experiments. Other modelling techniques can be easily substituted in place of linear regression modelling should they present better representation for a particular data set.

Experiments were conducted using the TOSSIM [5] simulator in order to examine the suitability of physical features to define the response of a communications solution in a network. The experiments examine three different solutions over a range of four main physical features: network size, node density, network diameter, link conditions and two secondary physical features: node density (standard deviation) and network diameter (standard deviation). A use case emulating a BEMS deployment is used for evaluation. Experiments are conducted in simulation using the TOSSIM [5] simulator. Each of the communications solutions tested use the Routing for low Power and Lossy networks (RPL) routing protocol within a BEMS deployment scenario. A range of communications solutions were tested each based on the RPL routing protocol. Different solutions and responses from the network are achieved by comparing different metrics with which the RPL protocol creates it’s routes. While the solutions evaluated in this article are based around the RPL protocol, the framework and testing process described is general enough to accommodate a wider set of solutions.

The main contributions of the article include:

- Introducing a framework for consistent evaluation, comparison and autonomous selection of communications solutions within a given deployment.
- Evaluating a set of physical features to determine which features have the greatest effect on the response of a communications solution in the network.
- Evaluating the use of linear regression modelling for predicting the best performing solution for a single evaluation metric in a network.
- Opening a discussion on a means for solution selection and communications context switching within the network during runtime.

The remainder of the article is structured as follows. Section 2 presents the state of the art and frames the work presented in this article within the literature. Section 3 describes the proposed framework with an overview of the frameworks modular architecture. Section 4, Section 5 and Section 6 introduce implementation details of each of the key modules within the framework. Section 7 describes how a dataset was collected from simulation for the purpose of evaluating the framework. Section 8 notes some discussion points and presents a critique of the framework and Section 9 presents the conclusions from the article and points to the direction of future work.

## 2. State of the Art

The IoT network domain is highly fractured. This applies to higher level standardisation [6] down to the direction, design and evaluation of new protocol and communications ideas. In order to have productive discussion it is necessary to narrow the focus to a more specific domain or use case. Much of the discussion will be focussed on IoT for smart cities, and in particular communications networks for BEMS. These systems incorporate building wide sensor and actuator networks primarily for collecting environmental data but increasingly for effecting change to the environment. Section 2.1 discusses the relevant communications solutions in this area. While each solution presents a unique method or performance profile the section stresses the inconsistencies in evaluation methods which leads to difficulties in comparing the solutions. Section 2.2 discusses the current techniques used to model and understand the underlying communications network. Many of these models focus on modelling the communication channel in IoT networks. Less work has been directed at modelling the performance of communications solutions as a whole in the network. Typically these performance measurements have been presented using empirical testing and evaluation of individual or sets of protocol in research papers directed at showcasing the abilities of a particular solution. We believe there is a significant absence within the literature with regard to the provision of standardised testing and overall performance modelling.

While nearly all papers evaluating newly proposed communications solutions agree that it is important to test the solution over a range of varying physical features of a network, there is no consensus or standardisation of evaluation methods. This article aims to shift the perspective from evaluation of communications solutions in isolated testing environments to a standardised evaluation process after which direct comparisons can be made.

### 2.1. A Range of Communications Solutions

It is widely published that there are a large range and increasing number of communications solutions available for the IoT networks [7,8,9]. Much of the previous recent work in the research community has been conducted in Wireless Sensor Networks (WSN) delivering alternative performance characteristics from the network for the needs of applications or tackling specific network conditions. Early work in this regard included Collection Tree Protocol (CTP) [10], LEACH [11] and SPIN [12]. Evaluation for the CTP protocol is predominantly based on packet reliability and was conducted over twelve open testbeds. While this is certainly considered rigorous testing it can be argued that the range of testing scenario does not exhaust the range of potential deployment scenario for the protocol. To give application developers an idea of overall performance other metrics must be included for evaluation. The LEACH protocol is designed for efficient use of energy using hierarchical routing techniques. LEACH is used as a comparison protocol for many other solutions throughout the literature. While this information is useful it is fragmented and difficult for use for direct comparison with new or other competing protocol. The evaluation of LEACH [11] is conducted on a single simulated environment. Energy efficiency is the single metric evaluated for nodes with network diameter, in terms of meters from a basestation, used as an independent variable. The evaluation of SPIN is similar with testing performed on a single testbed. Energy efficiency and useful reception rate are evaluated over nodes with varying network density. In both cases it is difficult to either directly compare or evaluate if the solutions are fit for purpose for a deployment with a specific set of requirements.

A number of recently introduced solutions such as TREnD [13] as well as a number of modified Routing for low Power and Lossy networks (RPL) protocols have added value to the IoT protocol discussion. There is a constant theme throughout the evaluation process in each of the papers, however, which echoes the issues discussed above. TREnD is examined using energy efficiency, latency and reliability as evaluation metrics. The testing is conducted in a single testbed making it difficult to predict how the protocol may perform in another deployment. Each protocol discussed is tested using a standalone environment or process ultimately making it difficult to compare solutions and select a solution for an application or deployment.

Recent efforts have seen a greater push towards protocol standardisation from both industry and academia. Amongst others IPv6 datagrams in Low Power and Lossy Networks (6LoWPAN) [14], RPL [15] and Constrained Application Protocol (CoAP) [16] are current protocol involved in standardisation. The three protocols constitute part of a possible communications stack proposed for smart city applications. These applications are envisaged to contain hundreds to thousands of sensors which are regarded as resources which can be accessed by the application. Handling Internet Protocol (IP) packets is regarded as essential for successful integration of IoT resources into current web systems. 6LoWPAN was developed as an encapsulation technique to allow IP packets to be passed through an IoT network without some of the unnecessary overhead of IP. RPL is a routing standard that is growing in support and adoption within IoT communities and projects. RPL uses a tree-like Destination Oriented Directed Acyclic Graph (DODAG) structure to connect all nodes to a single ingress/egress point for the DODAG. CoAP is a lightweight protocol which allows applications to request resources from the IoT using a HTTP-like RESTful interface. The standardisation of protocol is essential for the adoption of IoT technology but also for interoperability within and between IoT networks and we believe that this type of communications stack will form the basis of future deployments. It remains, however, difficult to cater for a full range of application requirements using a single defined stack. As well as this, maintaining a single stack philosophy may inhibit new protocol from being developed. The communications stack must remain malleable to change allowing layers to be swapped for competing protocol. Early attempts to achieve this [17] were not successful as competing MAC and routing protocol could not be compared effectively, leading to a position where the preferred protocol was not known. To this end, we believe that developing a standard for the evaluation process for protocol relating to smart cities is a crucial element required to accommodate new innovation while maintaining interoperability.

### 2.2. A Note on IoT Network Modelling

As a number of techniques exist for IoT network modelling this section acts as a brief discussion on the objectives of each alternate solution and highlights the differences between these techniques and that which is proposed in this article. A survey of modelling techniques is undertaken [18] detailing the type and scope of modelling available for IoT networks. The models range from modelling node components [19,20,21], to channel modelling [22], to whole system modelling [23]. The node based techniques aim to model all components to present a layer of abstraction from particular architecture or operating systems upon which applications can be designed. The modelling approach for the entire system presented in the survey focuses again on component analysis and how each component works together without eluding to how different components can effect the overall network performance.

A number of techniques use Markov chains to model the link and route behaviour within a network [24,25] with a focus on network performance. Kamthe et al. presents a fine grained model of links using a multi-level approach to model both long and short term dynamics on the links. Empirical data is used when building the model and the approach proves effective for modelling packet reception rates over a range of link factors including channel fading and packet frequency. Chiasserini and Garetto [25] present a network model based on Markov chains. The approach is agnostic of the routing protocol used but assumes a consistent protocol throughout. The system consists of three individual components: the sensor model, the network model and the interference model. The network model is based on a queueing network where each sensor holds a buffer of packets to be forwarded. The model does not consider the possibility of dropped packets focusing solely on energy cost and time delay through each queue. The model proves effective at determining time delay of packets through the network. However, as reliability is considered a vital evaluation metric, the proposed model cannot be used for direct comparison of communication solutions. Analysis and modelling of Media Access Control (MAC) and routing protocol interaction in IoT networks is also achieved using Markov chains [26,27]. The results from these papers focus on further advising of the best routing parameters to achieve the best reliability and latency in the network. The experiments and analysis are based on the DODAG network structure also used in the RPL protocol. The analysis performed in these papers are quite specific to this particular network structure and cannot be applied to a wider range of protocol.

A fundamental difference exists between the modelling suggested in the literature and the modelling undertaken in this article. The literature describes a number of techniques to determine a generic model of an IoT network usually based on component, link characteristics and modelling forwarding mechanics. The models presented are useful for higher level application development or even improving simulation precision for IoT networks. The models presented focus on modelling the mechanics within a network using particular assumptions about MAC and routing protocol or network structure. This article describes a method to determine a performance model for a range of communication solutions based on the physical characteristics of the network. The focus is placed on modelling how a solution performs as a whole in a network, rather than modelling the mechanics exhibited within the network. The benefit of which is to directly compare otherwise disparate solutions within the same network conditions. This can directly advise on a communications solution for a deployment without the need for extensive testing or network specific knowledge. While some of the presented models do perform experiments, all models presented in the literature are based on theoretical reasoning where as the models presented in this article are derived explicitly from simulated testing data.

## 3. A Framework for Evaluation and Modelling

As well as presenting a framework for consistent evaluation of communication solutions, it presents a system for autonomous monitoring and solution selection within a deployment. The framework aims to reduce the need for extensive knowledge and experience in IoT communications when developing applications for the IoT platform. It reduces the need for manually testing a network when choosing an appropriate communication solution for a deployment. By creating a layer of abstraction from the network the framework assumes management of the communication layers allowing a developer to build applications without exposure to these lower layers. The developer simply provides the requirements desired for the application and the framework implements a solution which best fulfils these requirements. The framework is able to monitor the network environment and choose, from a library, the communication solutions which will best facilitate the application requirements within the given environment. The solution is chosen automatically without extensive testing on the particular deployment and without human oversight. A key aspect of the framework lies in how it can ascertain the response, in terms of network performance, of a solution given a certain environment during runtime.

**Figure 1:**
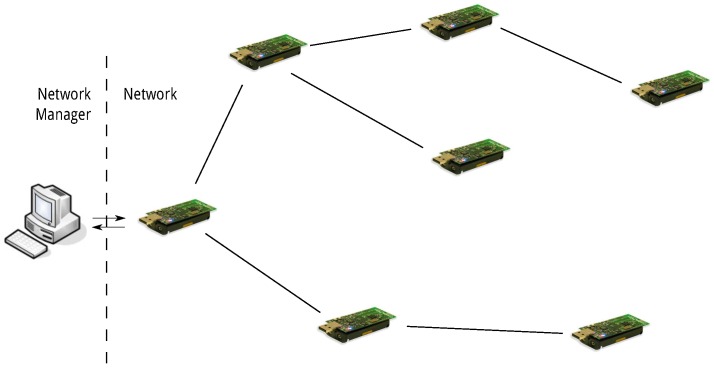
The *network manager* residing on a device capable of higher level processing power either on or connected to the root of the network.

The framework relies on pre-built models to establish the expected performance of a solution in a given environment. These models are based on data gained from prior testing of the solutions, which may not necessarily have been conducted on the environment in question. Each communication solution is first tested using equivalent evaluation methods in order to determine how each responds over a range of network characteristic features. This implies that the same evaluation metrics and network characterisation features must be used for all testing procedures. From this data, a model is built for each solution that predicts the performance, in terms of the evaluation metrics, of the solution within any deployment from which these features can be measured. In this article a set of the networks physical features: scale, density, size, and channel quality are used as as the basis of the performance model. A discussion on these features and their effect on the response of a solution is presented in Section 4.1. The benefit of these models allows pre-testing of the solutions which can then be used in real time over a wide range of deployment types without explicit testing in the specific deployment. A network manager dictates the necessary tasks required for network control. This includes network probing to ascertain the physical features of the network, comparing expected response with application requirements and controlling network state. These tasks require additional processing power. The network manager resides at the root of the network as shown in Figure 1.

**Figure 2:**
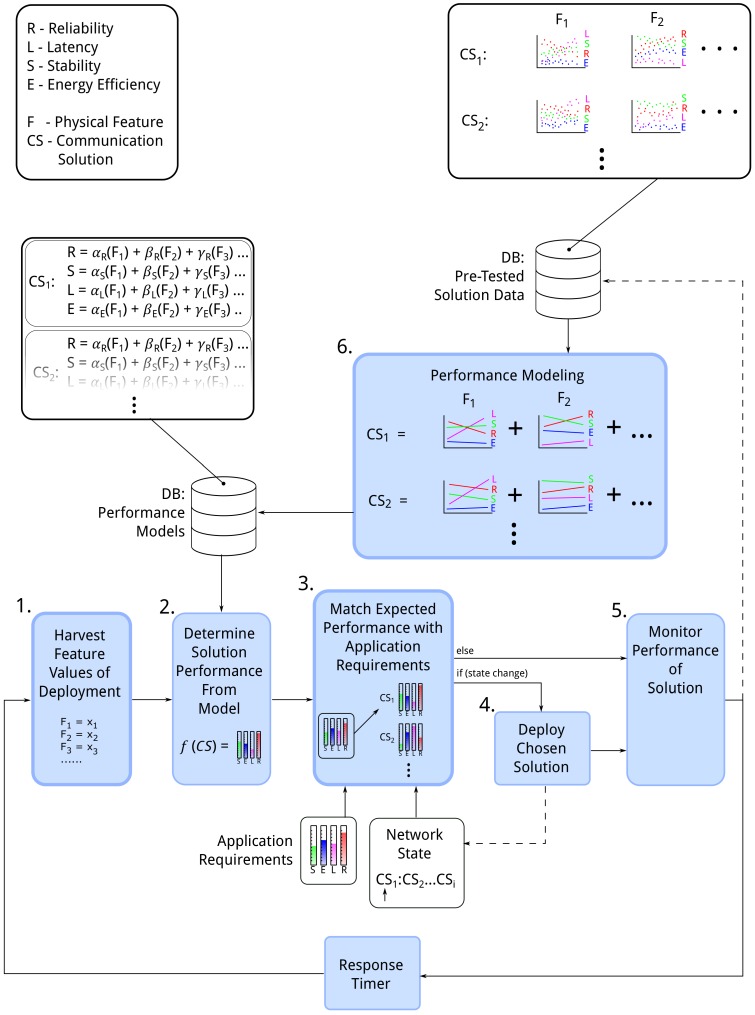
Functional block diagram showing the component relationships for the framework.

It is important to note that the framework takes a macroscopic perspective of network control. Unlike other network control systems it does not engage in route dynamics and repair or indeed network level scheduling. Instead the framework uses and implements entire protocol changes to the network to best fulfil application requirements. A functional block diagram showing work flow and processes undertaken within the framework is shown in Figure 2.

The processes in the framework include:

**1. Harvesting features from the deployment.** This process includes probing of the network and gathering of physical features to the network manager most often at the *root*. The process begins with a single packet sent by the network manager to all nodes in the network identifying the desire for physical network information. Each node to receive this packet sends a beacon to its neighbours requesting a reply packet. From the reply packets the node generates a neighbour list including the physical channel indicator. When the information is collected at the network manager, it will build a network topology from which it can extract those features necessary to compare solutions using their performance models.

**2. Determining expected solution performance from the model.** The physical feature values extracted during harvesting are used when comparing communication solutions. Pre-built models for each solution are interrogated using the current feature values. A set of expected performance metrics are then generated for the communication solutions.

**3. Matching expected performance with application requirements.** The expected result for each solution will be returned from the models giving a value for each desired metric. In order to choose a solution they must be compared to find the most appropriate selection as per the application requirements. The appropriate solution is unclear, however, when a comparison is made over each of the metrics. For example: *solution 1* returns a reliability of 90% and a latency of 0.23 s while *solution 2* returns a reliability of 81% and a latency of 0.05 s from the given network. If the applications requirements state that reliability is valued as twice as important as latency it is unclear at first look which solution is the most appropriate for use. Is a 9% gain in reliability worth a 0.18 s loss in latency even with the importance weighting? To solve this each solution must be provided a single score which reflects the expected response and its adherence to the desired requirements. From this score a clear winner can be chosen for use in the network. Section 6 presents a statistical approach to achieve this.

**4. Deploy chosen solution.** Once a solution has been chosen that reflects the network requirements desired by the application it must be deployed to all nodes to take effect. The network manager maintains the state of the network with regard to the currently implemented solution. If the chosen solution identifies the currently implemented solution no further action is necessary, otherwise the network manager must take steps to implement a new solution. Once a solution has been implemented the *network state* is updated. Two techniques are discussed in Section 6.3 which may be used to implement the chosen solution:

- Reprogramming each node with a new binary should the network require a change of solution. This solution carries extensive communications overhead.
- Switching between solutions with the implementation of each solution present on all nodes at all times. This solution requires increased memory overhead and is less flexible to the introduction of new solutions.

At the outset of the network deployment a predetermined solution from the possible solutions list is deployed in the network for the purpose of network probing.

**5. Performance monitoring.** Elements of performance monitoring may or may not be available depending on both the application and the network infrastructure. Essentially this process involves observing the network to determine the performance in terms of evaluation metrics. If these metrics are available they can be fed back into the solution data database for refined modelling. Should the deployment involve debugging infrastructure or maintain node time synchronisation over the network many of these metrics such as packet reliability, latency, energy efficiency and route stability can be monitored.

**6. Performance modelling.** Determining effective models for use in solution comparison is integral to the frameworks success. Models are built from the solution data database. The modelling process is discussed in detail in Section 5.1. The modelling approach favoured in the presented framework involves determining the performance of a metric using linear regression over the range of features.

The framework is built so that any facet of the system may be replaced to achieve better performance. One simple example would be to update the performance modelling module should further research dictate another modelling method to be more effective.

## 4. Physical Features and Response Characteristics

It is clear from the literature within the field that physical features such as network size and density are regarded as important features which shape the response of a solution in the network as they are commonly used as independent variables for evaluation. This has been considered a priori to experiments and as a result no research has been directed at determining how much of an effect individual features have and if this effect is uniform over all communications solutions. Experiments conducted in this article address how each physical feature effects the ability of a communication solution to provide a level of Quality of Service (QoS) for an application while presenting data to determine the suitability of using a set of physical features as a means to model the response of a communications solution.

The modelling process plays a major part in the framework described in Figure 2. The framework is highly dependent on how well a network can be modelled from the feature set. A number of challenges arise, however, when attempting to perform experiments which model an IoT network for this process.

- Many physical features exist which may effect the response of a solution. The goal is to build the simplest model, with the fewest features. This set should also consist of the physical features which effect the greatest influence over the response. Determining this set is in itself challenging for two reasons: (i) Each communication solution reacts uniquely to the physical features of the network. Defining a set that incorporates the main influences for all solutions becomes more difficult; (ii) Removing features from the set may leave certain response types not covered by the model.
- Even with extensive testing on a given deployment it remains difficult to guarantee a level of QoS for an IoT network. Determining in absolute terms how a solution will perform on a particular deployment remains difficult even with effective modelling.
- The chosen features must be those which are easily obtained from the network in order to minimise the required testing on each network before deployment.

The physical features used for testing are carefully chosen to ensure they encapsulate the main proponents determining the network response for each communication solution. In order to do this a number of different solutions, based on the RPL protocol, are tested over a range of physical features. The extent of the effect each feature plays in shaping the response is evaluated, determining which features are most prominent.

### 4.1. Defining the Features

The physical features chosen to model the network response are pivotal to the success of the proposed performance model. A significant limiting factor applies to any feature which may be considered for use within the model. This states that any feature must be easily harvested from the network to minimise the amount of testing on each deployment. A further refinement is made stating that features must be available to simple packet probing contained within the networks own infrastructure, negating the need for additional debugging tools. This allows the possibility of a fully functional feature set to be garnered from a connected remote location, further reducing the cost of testing a deployment. With this constraint in mind, a set of features is defined as consisting of:

- Primary
  1. Size of network (number of nodes in the network).
  2. Network density (average over node densities).
  3. Network scale (average over individual node diameters).
  4. Channel quality (average link quality over whole network).
- Secondary
  1. Network density (standard deviation).
  2. Network scale (standard deviation).

**Figure 3:**
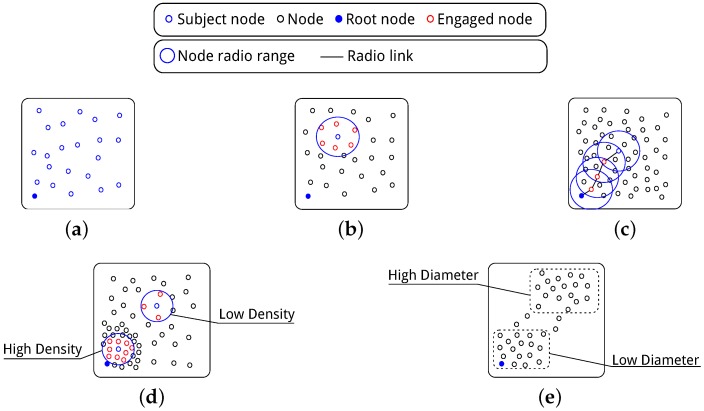
Illustration of the manifestation of the physical features in the network. (**a**) Size is 22: Total number of nodes that constitute the network; (**b**) Density, 6: The number of nodes within communication range; (**c**) Diameter, 4: Minimum number of hops necessary to reach the root; (**d**) Illustrating a high standard deviation in terms of network density. This scenario can occur when a number of highly sensed environments exist with sparsely sensed environments within the same network; (**e**) Illustrating a high standard deviation with regard to network scale. This occurs commonly within buildings when nodes in geographically spaced rooms exist on the same network.

While this does not constitute an exhaustive set of features, it fulfils the main constraint and provides results which validate the suggested framework. Figure 3 illustrates these features with further discussion on each provided.

**Figure 4:**
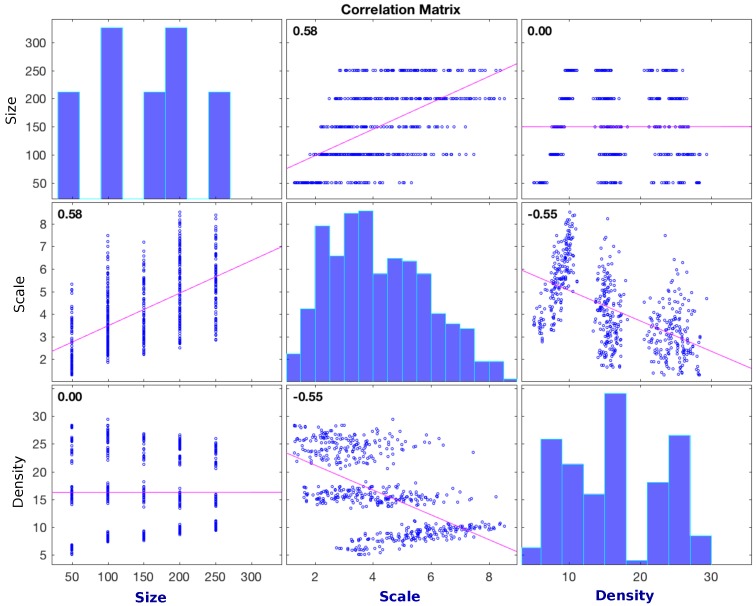
Relationship between physical features in simulations.

The *size*, *density* and *scale* are linked, with each feature dependent on the other two. For example, if density and network scale are both high, the network size must be large. A high network density with low number of nodes would indicate a low scale. The dependency is defined in Table 1 outlying the Pearson correlation [28] between each variable and displayed in a correlation matrix in Figure 4. Both the table and the graphs imply a strong correlation between density-scale and scale-size. These are expected results from the experimentation phase. During simulations the main variables used were network size and physical area of the network. To asses a range of densities of a given network size the network area is varied dictating a change in scale. A change in the size of the network also dictated a significant change in scale as denoted by the *p*-values. The points in the graph represent the features of each of the 559 generated network variations used in the testing phase described in Section 7. The graph includes the networks from both training and test data sets.

These facets also represent the main aspects of the network topology. Depending on the application, the size can greatly effect the amount of packets sent in the network. Density effects channel contention while scale effects hop count in the network with deviation in both greatly affecting latency, reliability and energy efficiency in the network. Taking the average density and scale over the whole network gives a good insight into the network topology but does not present the full picture. The scenarios shown in Figure 3d,e are common and cannot be expressed using node averages. The standard deviation then becomes important to determine these features. A taxonomy of the network composing of these features is easily obtained by each node sending its list of neighbours to a centralised point.

*Channel quality* is of importance to the Packet Reception Ratio (PRR) on each link. Increasing the average channel quality across the network will increase PRR dramatically affecting the evaluation metrics. Taking an average of the channel quality of all links to a node may not fully represent the quality of the channels that actually get used within the network and as a result may not fully represent performance. This can occur when many “fringe” neighbours appear to a node, none of which are chosen for communication. A more accurate description of the used channel can be achieved by also using the best quality channel available to each node. A number of measurements are used to assess channel quality including Link Quality Indicator (LQI) and Received Signal Strength Indication (RSSI). Such measurements are available using the CC2420 [29] chipset typically used for IoT communications.

### 4.2. Harvesting Physical Features from the Network

**Figure 5:**
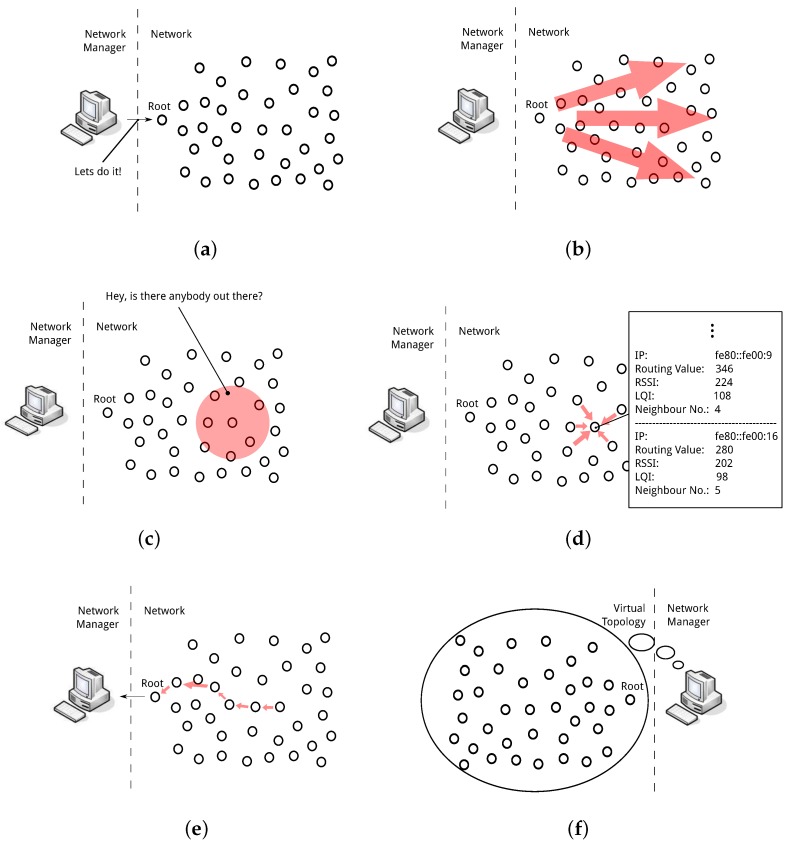
Information gathering process instigated by network manager. (**a**) The network manager sends a message to the root of the network instigating the information gathering process; (**b**) The root relays this message to all nodes in the network; (**c**) Each node in the network sends out a beacon requesting information from its neighbours; (**d**) The neighbouring nodes send a reply and a neighbour list is generated with physical link information included; (**e**) Each node in the network sends its neighbour list back to the root of the network and this information is then passed to the network manager; (**f**) The network manager maintains a virtual topology with the network information from which it can extract the physical features.

Harvesting information about the network allows the framework to learn the state of the physical deployment with regard to the features necessary for solution comparison. The network manager initiates a probing regime in order to interrogate the deployment. Figure 5 shows how this process is performed in the network.

The process is initiated by the network manager by sending a message to the root. On receipt of this message the root must inform the entire network. The root may use the structure of the current communication solution or a flooding technique to proliferate the message to each node in the network. Each node then sends a beacon within its neighbourhood which requests a reply from each neighbour. Addressing a particular node when replying incurs additional communication overhead particularly in dense networks but increases reliability on information retrieved. With the replies received a node will generate a list of the physical features in its neighbourhood. When a node is confident it has received all replies from the network a packet is sent to the root containing the information it has garnered from its neighbourhood. The root passes this information to the network manager from which it can build a virtual network topology from the local physical features. Network wide physical features can be obtained from this virtual topology.

**Figure 6:**
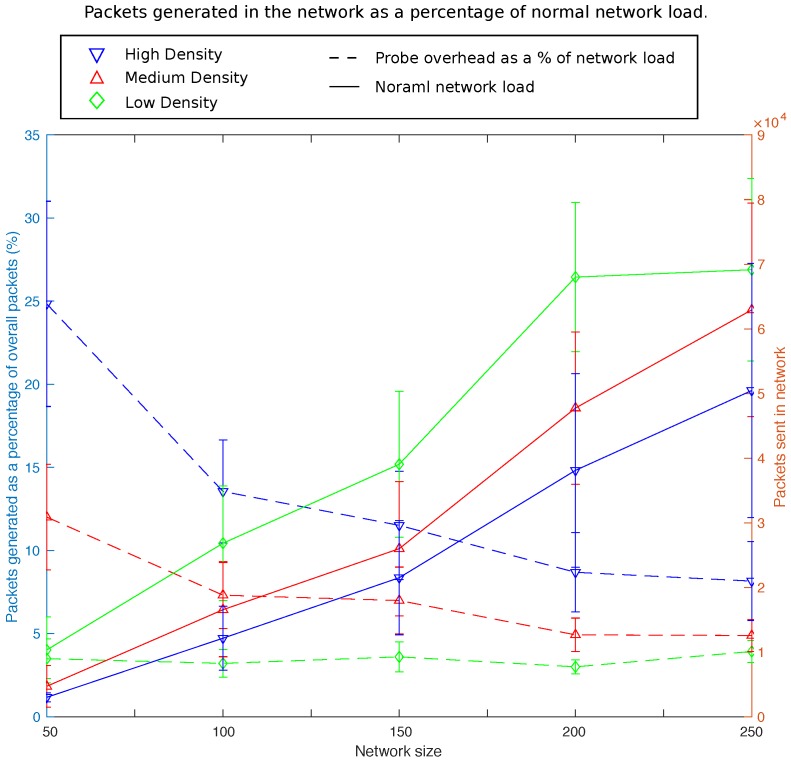
Mean and standard deviation for the maximum percentage load incurred using the probing technique over normal network load.

The information gathering process is initiated at the outset of the network to first establish which communication solution to use within the deployment. The network manager then initiates this process in a periodic manner throughout the network lifetime to remain reactive to any major changes that might occur within the network. As the framework takes a macroscopic perspective on network control the network manager does not need to respond to small fluctuations within the network. These changes are handled by the more dynamic communication solution deployed in the network. Large changes to the network, however, may necessitate a change of communication solution. Large changes to the physical network occur much less frequently allowing less frequent probing by the framework. Using the same BEMS scenario as described in Section 7, an evaluation of the additional overhead incurred via the information gathering process is undertaken. The results in Figure 6 show the additional overhead incurred during information gathering assuming this process is initiated once every running hour. The data shown in the graph is taken from simulation during the testing phase described in Section 7.

The figure shows results over a range of network sizes and densities. In general a higher load is observed in the network at lower densities. This is in part due to larger number of retransmissions in the network as well as observing a larger diameter compared to more dense networks of similar scale. The frameworks probing process shows heavier burden on the network at higher densities. This is due to the addressed reply policy employed by the framework. In dense networks each node will receive many beacons and reply to each of them individually increasing overhead. The data collection overhead as a percentage of the overall load is reduced as the size of the network increases. This is due to the wider diameter of the network at higher scale. While higher network densities will have a large knock on effect on the collection process, the diameter of the network has a lesser impact on the data collection process than that on normal network load.

It is important to note that this is the maximum overhead which the network may incur as a result of probing by the framework. There are many ways this overhead can be reduced within the network. Should a communication solution maintain a full list of neighbours including the physical link metrics for neighbours, steps 5c,d from Figure 5 are no longer required, significantly reducing overhead. Allowing local physical information to piggyback on data packets can further reduce network overhead.

The information gathering process plays a vital role in the autonomous framework. This does however incur additional overhead to the network. Depending on how dynamic the network must be to change, this overhead can be more or less significant. Once all the information from the network is gathered, the network manager can extract all physical features necessary to complete the models and calculate expected performance levels for each solution. Matching the correct solution to the application preferences, described in Section 6, is then necessary.

## 5. Modelling the Communication Solutions

Modelling the performance of each solution is for the purpose of performance prediction. Basing a model on features that are easily obtained from a network deployment is key to reducing the amount of testing required to characterise a deployment. The model can be used during runtime to compare solutions and advise on the best communication solution for a given set of requirements in a particular deployment.The goal of this is to reduce the need for extensive knowledge for application developers and to greatly reduce the amount of testing required on individual networks before deployment.

### 5.1. Modelling an IoT Network Using Physical Features

In this section a procedure is presented to model the IoT network based on its physical features with the aim to give insight into the best solution for a particular deployment. A model for predicting network performance is built for each communication solution based on these physical features. The model is then tested to determine if it can be effectively used to compare communication solutions.

A dataset taken from the testing procedure described in Section 7 is used to build and evaluate a model that describes the response the solutions in the network. Experiments are conducted using three network solutions each based on the RPL routing protocol. Each solution differs in choice of routing metric with Expected Transmission Count (ETX) [30], Expected Transmissions with Neighbourhood Heuristics (ETX-NH) [31,32] and Expected Transmission Time (ETT) [33] used in the comparisons. Each solution is tested over a range of the physical characteristics used to describe the network. The dataset is split into two distinct sets, a training set and a test set. The training set is used to train and build a model that describes the response of the communications solution. The test set is used in the evaluation process when determining the accuracy of the models. Both the training set and test set contain equal number of data points spread over the whole range of each physical feature. This ensures the model is built from and tested over the widest range available in the dataset. The results suggest that IoT network communications can be modelled using key physical characteristics of the network.

Using multiple linear regression techniques over training data set a model is built that represents the response of a communications solution in the network. The eventual model will present heuristics which inform the developer. The heuristics can be used in the form: *Solution A* shows *effect on metric B* for *physical scenario C*, e.g., *ETX* shows *superior packet reliability* for networks with *network radius greater than 5 hops*. In summary the following contributions are made from the modelling process:

- Define a set of features of a physical network that are best disposed to characterise the response from the network for a given communication solution.
- Build a model for each communication solution which presents the trends seen in the network response, in terms of latency, reliability, energy efficiency and stability, as each physical feature changes.
- Test the model and compare communication solutions so that the findings can be used to direct decision making in future deployments.

Section 5.2 introduces the techniques used to model the network using physical feature data set and provides an evaluation of the models accuracy.

### 5.2. Model Performance

The model [34] is built with the statistical package R [35] using the data taken from simulation. Multiple linear regression is used to build each facet of the model. Linear regression modelling is used for two reasons. The figures presented in Section 7 show visually how the data produced from the experiments fit a linear trend for each of the evaluation metrics with regard to the changing physical feature. This observation is more acute however for some solutions, ETX and ETX-NH, than for others, ETT. In all figures for reliability and stability, ETX and ETX-NH show a linear trend with a comparatively low standard error while ETT shows wider value dispersion and higher standard error over the range. In general the ETT solution shows wider dispersion of values for many of the physical features and shows wider standard error from a linear trend for the reliability, stability and efficiency evaluation metrics, though the data does show a general linear trend throughout. Liner regression modelling represents a simple modelling technique which is well suited to demonstrate the purpose of solution modelling within the framework. For each solution, least squares linear regression is used to model how each evaluation metric responds over the set of physical features. The set of physical features are used as the predictor variables for each of the models, with each of the evaluation metrics acting as the response variables. For example, reliability is used as a response variable with predictor variables network size, density, scale, link conditions, size (standard deviation) and scale (standard deviation). Each communications solution will have separate model built for each response variable resulting in twelve individual models for the dataset. A separate model is required for each evaluation metric as the it is important at this stage to give an individual result for each of the metrics. Applying a single score for a network solution would assume that all applications value each of the metrics equally. A method to combine the evaluation metrics into an overall score is discussed in Section 6.1. Separating the modelling and the scoring process in this manner allows the pre-built models to be exported for any application without re-building. As the physical features drive the predicted result for each of the models, it is interesting to note which physical features have the largest impact on each result. A T-test is performed determining the features that constitute a significant role in shaping the response for each metric (response variable). Table 2 highlights these significant features.

The table shows how each communications solutions response is shaped using different features. The table returns some expected results, such as the importance of scale, size and the communication channel in determining the response for nearly all cases, but also offers other insights into the dynamics of the evaluation metrics with respect to the features. Scale (standard deviation) has a large impact on ETT response while having less effect on ETX and ETX-NH. While scale is generally important in determining the response for all the evaluation metrics it has less of an impact in determining the stability of a communications solution.

These individual models are used to determine whether the proposed approach can reliably determine the correct solution to use in a network given a set of requirements. In order to achieve this the problem is broken down into a set of smaller problems, asking whether an ordered list can be determined of performance capabilities per metric for each solution, i.e., for any given set of physical features the model determines the order of the solution performance from best to worst for each evaluation metric. A model is built for each solution and evaluation metric from training data. A test data set is then used to examine the accuracy of the models. Accuracy is measured with regard to how well the model can predict the best (1), middle (2) and worst (3) performing solutions in terms of reliability latency, efficiency and stability for a given scenario. Table 3 shows how accurate the model is at determining the placement for each metric on test data obtained from the TOSSIM simulator.

The model is evaluated using percentage accuracy, precision, recall and F1. The percentage is measured as the percentage of true positives predicted by the model. Precision is measured as a ratio of the true positives against the true positives and false positives. Recall is measured as a ratio of the true positives against the true positives and false negatives. F1 is shown in Equation (1).

(1) $$F1=2\times \frac{\mathrm{precision}\;\times \;\mathrm{recall}}{\mathrm{precision}\;+\;\mathrm{recall}}$$

With regard to the application context the precision rate and recall will relate to the ratio of how often the system can correctly choose the required solution compared against how often it chooses the incorrect solution. The F1 score shows a weighted mean between the two scores The precision and recall rates are closely bound and in general show lower accuracy compared to the percentage score. The percentage accuracy precision and recall all follow the same general trend across the tests. The evaluation presents mixed results. The model achieves highest accuracy when predicting comparative performance with regard to latency and reliability reliability. prediction accuracy is not uniform over all evaluation metrics, with accurately predicting efficiency proving most difficult. Accuracy of prediction reaches as low as 50.43% in some categories, with recall as low as 0.4032 and precision as low as 0.4360. These lower scores are not acceptable for a production system when predicting between three solutions. Despite some mixed results the model presents a positive outlook for the prospect of using the physical features of the network to compare communication solutions to find the best fit for a set of requirements.

**Figure 7:**
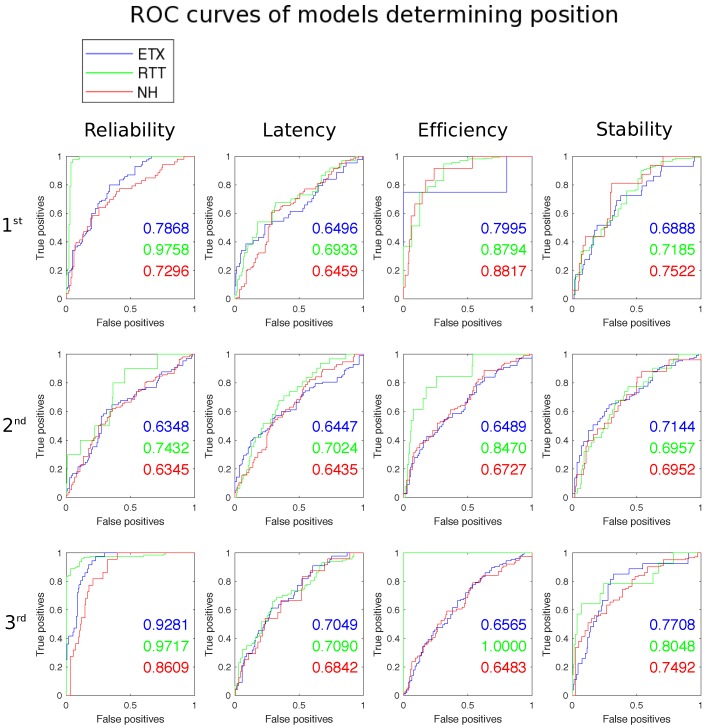
ROC curves including AUC value when determining the rank position for each solution for each evaluation metric.

A Receiver Operating Characteristic (ROC) graph is used to determine how well the physical features used in the modelling process are predisposed to distinguishing the rank of each solution for each evaluation metric within the training data set. Figure 7 shows the curves and Area Under Curve (AUC) for each. Each curve gives a value for a binomial class distinction. It shows how well the predictor variables can determine if a solution performs in a given rank, or not in this rank. For example, a single curve will specify how well the predictor variables distinguish between experiments where ETX performed in 1st place and those where ETX did not perform in 1st place. The AUC gives a single value metric between 0 and 1 with a higher value denoting a better predictor variable set for such a class distinction. The models return an AUC value between 0.63 and 0.97. The physical features used as predictor variables used in the models prove more adept at distinguishing ranked places for the ETT solution with near perfect classification achieved for reliability (1st and 3rd place) and efficiency (3rd place) although somewhat surprisingly does not show as high AUC values for latency given the stark delineation of rank. The predictor variables perform well classifying reliability, efficiency (1st place) and to a lesser extent stability (1st place), however, are less well predisposed in classifying the placed ranks for latency.

## 6. Matching User Preferences to Network Response

Selecting the best communication solution given the application preferences is important for delivering the most efficient network for the application. This involves matching the preferences to the most suitable solution given the derived expected results from the model. Even with effective modelling, matching the response to application preferences is not a trivial task. The quality of a communications solution is defined over a number of unlike variables and creates difficulty when comparing these unlike variables for decision making. The solution quality is measured with metrics such as reliability, latency, efficiency and stability with each metric calculated with different units. In order to compare the communication solutions each solution must be given a single score which represents all the evaluation metrics. Creating this composite score requires some of the tenets of Multi Criteria Decision Analysis (MCDA) [36]. First a structure is put in place by which the evaluation metrics can be standardised to like variables for combination. These variables can then be scaled before adding a weight based on the application preference. When the variables are combined a clear winner can then be selected. Section 6.1 describes this process while Section 6.2 presents an example using the models created in Section 5.1.

### 6.1. Combining Evaluation Metrics for Comparison

Scoring the evaluation metrics is integral to the eventual decision. This involves standardising the disparate criteria into like variables which can then be combined. While scoring the criteria can be a domain specific problem resources are available to guide a scoring structure [37]. Two techniques are generally available for attributing a score to individual criteria: local and global scaling. Local scaling involves setting up an arbitrary scale, from 0–1 or 1–100, and attributing scores for the criteria within this scale. This is generally used for qualitative data. Global scaling involves bounding the scale between two extreme possible values of the criteria. For example, in a test set the lowest latency for an experiment is given as 0.02 s while the highest latency is 0.34 s. These values become the bounds on the scale. If a solution returns a value of 0.02 s it is given a standard score of 100 (lets say), while a solution returning a value of 0.34 s will be given a standard score of 0 and a value of 0.16 s is given a score of 50.

A specific scoring structure is created for the evaluation metrics considered during testing in Section 7. A form of global scaling is used in the scoring mechanism. The scoring structure is based around the mean of the criteria over the expected performance results from all communications solutions. To create a standard score for a communication solutions reliability value, the mean of the predicted reliability values for each of the communications solutions is calculated. The criteria score for the communications solution is taken as the percentage difference from the mean. This is formulated in Equation (2). This produces a positive score for criteria with a value better than the mean and a negative score for criteria with a value lower than the mean.

(2) $$\mathrm{Criteria}\;\mathrm{Score}=\frac{x}{\bar{x}}\times 100-100$$

Scoring the evaluation metrics as relative to the mean is an effective way to present likewise disparity of values over disparate scales. It is still necessary, however, to apply additional scaling and weighting to the criteria scores before they can be summed to create a solution score.

#### 6.1.1. Scaling Variables

**Figure 8:**
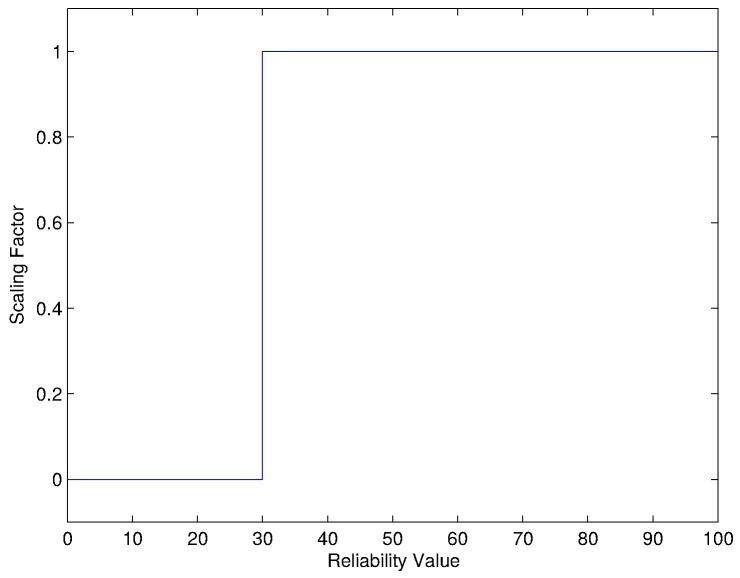
Scaling factor for the reliability criterion.

Scaling variables is performed for two reasons: (1) to add contextual information with regard to the criteria and (2) to correct an imbalance introduced while scoring. Adding contextual scaling is domain specific and is applied to the criteria on a case by case basis. When considering the criteria involved in the current example, there is a rationale to apply a scaling factor to the reliability criterion. One can argue that a reliability of less than 30% is of little to no benefit to the network. Figure 8 shows a scaling factor can be added to remove any additional score from the reliability criterion to the solutions overall score should the reliability value be lower than 30%.

Correcting an imbalance is required if the scoring scheme is imbalanced with a single or set of criteria overshadowing the overall score. The scaling is used to reduce the effect of the criterion that would otherwise exaggerate the difference between solutions. This imbalance can occur where the scale for scoring a certain criterion has a much wider range than the others. A simple weight correction can be applied to redress this imbalance. This is usually in the form of a multiplicative scaling factor. A weight correction is applied to both the efficiency and stability criteria seen in the example in Section 6.2.

#### 6.1.2. Weighting by Relative Importance

To influence the solution scores to represent the desires of the application a weighting regime is imposed. This will add a weight to each of the criteria as per the needs of the application. Should an applications dictate a high requirement for latency, a weight will be applied increasing the effect of latency on the overall score. The solution with the best latency will thus enjoy an improved score over the other solutions. A weighing scheme using relative importance is used on application preferences. A relative importance weighted scheme is easily achieved with quantitative value criteria. The application simply places an importance in terms of a number from 1–100 on each of the criteria. If all criteria are of equal importance the application could place an importance value of 100 on them all. The weighting factor is derived directly from the importance value with an importance value of 36 receiving a weight of .36 and an importance value of 78 receiving a weight of 0.78 and so on.

The result of these processes is a set of scores which are representative of the quality the solution provides but also reflect the application goals within the network. From these scores a clear winner is easily chosen. The winner is the communication solution which is most likely to achieve the best fit with the application requirements within the current environment.

### 6.2. Combining Evaluation Metrics: An Example

To further illustrate the scoring process involved when choosing a suitable communications solution an example is provided. This example demonstrates the criteria scoring, scaling and weighting involved in decision making using data and models presented in Section 5.1. First, a hypothetical set of physical attributes for the network are defined (Table 4).

Using the models an expected value for each of the evaluation metrics for all solutions is determined. After applying both the scoring and scaling structure a score for each criteria is available for preference weighting. These scores are shown in Table 5. As latency, efficiency and stability are minimal metrics, a positive score is required for values lower than the mean and visa versa. Equation (2) is adapted to facilitate minimal metrics giving Equation (3).

(3) $$\mathrm{Criteria}\;\mathrm{Score}(\mathrm{minimal}\;\mathrm{metric})=100-\frac{x}{\bar{x}}\times 100$$

The imbalance due to the extended value ranges for both efficiency and stability are corrected by adding a scaling weight of 0.5 to the score. The scaling scheme used for the reliability criterion does not effect any of the results in this example.

Table 5 shows the criteria scores before a weighting is applied. The weighting is applied in direct correlation to the application preferences. After the weighting is applied the scores can be added to determine a winner. A different winner can be determined depending on the preference of the application. Table 6, Table 7 and Table 8 show a number of preference models and winner outcomes of each. The preferences are detailed in Table 9.

The preferences are used as a multiplicative weighting factor on the criteria scores. After weighting the criteria each of the criteria scores are summed to create the solution score. Table 6 shows the solution scores which advise best solution for the preference set A. This preference set holds all criteria at equal importance. The ETX-NH solution is a marginal winner with this preference model due to a strong performance for reliability and stability. ETX is the chosen solution for preference set B. Increased importance is placed on both reliability and latency with little importance placed on stability and efficiency. Despite the increased importance focusing on latency in preference set C, ETX is chosen as the best solution. This is due to the poor performance shown by the ETT solution in both stability and efficiency.

### 6.3. Deploying Solution to the Network

Switching between solutions during runtime allows the framework to direct and carry out network changes where necessary achieving full network control with complete autonomy. There are two possible techniques that can facilitate this without requiring either network downtime and human involvement, or additional hardware infrastructure in the network:

- Switching between solutions, each present on all nodes at all times using a **single binary**.
- Reprogramming the **running binary** on each node in the network as necessary. The new binary contains the chosen communications solution to be used in the network.

**Figure 9:**
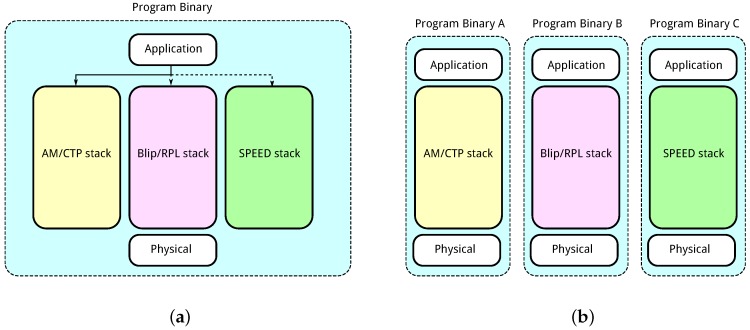
Single binary and multiple binary program solutions. (**a**) Program binary containing multiple communication solutions; (**b**) Multiple program binaries containing a single communication solution each.

Figure 9 shows a representation of single binary and multiple binary solution programs. Each node in the network can run a single binary at any given time. Both solutions have associated benefits and disadvantages with their use.

**Single binary.** This technique requires all solutions, that may be used by the framework, to be present on a single binary which is run on all nodes in the network. The binary will then contain one communication solution which is currently being used and a number of other solutions which are not in use as per Figure 9a. A node can change between solutions by passing messages between modules on the node itself. Switching between solutions requires little communication overhead or network downtime. When the network manager wishes to change communication solution it simply sends a message to all nodes in the network. The nodes can then switch between the onboard solutions. An additional framework is necessary to facilitate switching between solutions in a single binary. The main disadvantage of the single binary technique, however, is the increased memory footprint of the program inducing additional memory requirements on the nodes in the network. As more solutions are added to the program, the memory requirements are increased which may prove prohibitive for many node types.

**Multiple Binaries.** A more traditional approach involves using a single communication solution per program binary as shown in Figure 9b. Each program binary has a memory footprint capable of fitting most node types. When a change of solution is required, a new program binary is distributed throughout the network. Each node switches the running binary to the new binary implementing the required communication solution. This allows a greater number and disparity of solutions to be available for use by the framework in the network. When the network manager chooses a solution to implement it must compile the application and chosen solution into a single binary which can be run on the node. The binary is then distributed through the network using a source code distribution technique such as deluge [38]. Distributing the binary program to each node in the network incurs heavy communication overhead.

The single binary technique is beneficial for examples such as in Section 7. This example employs three unique solutions based on a single communications stack reducing the memory requirements significantly. Changing between solutions requires only a single message sent to each node to inform the change. The memory capabilities of nodes in the network may prove restrictive when employing a more diverse set of communication solutions. The multiple binaries solution is necessary when the single binary solution is infeasible. This incurs additional communication overhead but facilitates the use of a large range of solution options.

## 7. Testing

In order to validate the proposed framework and determine if physical features are fit for the purpose of modelling the response of a network a number of experiments were undertaken. The purpose of the experiments was to provide data to build a network response model for a number of different communication solutions. The goal was to present an easy form for comparison between the communication solutions. The response of the network is measured in terms of four evaluation metrics: reliability, latency, efficiency and stability. Reliability is the measure of how many sent packets get received at the root as a percentage. This is calculated as the ratio of the total number of packets received (removing duplicates) at the root node over the total number of packets generated (not including forwarded packets) across the network during the experiment. Latency is an average over each node in the network. The nodes latency is the average time a packet, originating from that node, takes to reach the root. Efficiency is measured as the total number packets sent through the network during the experiment. This includes all application packets forwarded through the networks as well as packet retransmissions. The number of packets sent is used as a proxy for energy use within the network as the energy consumed by transmitting and receiving packets far exceeds other energy considerations [39]. An energy efficient routing protocol will route a packet to the intended destination with the fewest number of packets. Stability is measured as the number of route changes occurring in the network. This metric is often described as route churn. The metric is calculated as the total number of route changes made in the network over the duration of the experiment.

Experiments were conducted on the TinyOS SIMulator (TOSSIM) simulator [5] with TinyOS [40] used as the sensor platform for each. The simulations use an array of micaz nodes. TOSSIM uses noise modelling with Closest-fit Pattern Matching (CPM) [41] to model the packet reception rates at receiving nodes. The links can be varied by introducing increasing levels of noise on the channel. A noise trace derived from empirical testing can be introduced to simulate dynamic link conditions. Each experiment was run in simulation for a network time of one hour with each node in the network sends a packet to the root each minute. This replicates a simple BEMS scenario for building environment monitoring. This provides the basis of many BEMS applications. Each experiment is conducted on a distinct topology generated for use within the TOSSIM simulator. Nodes in the simulated network are randomly distributed within a unit square with each node having at least one usable link to another node. This is only the case, however, in perfect channel conditions. Introducing noise to the network may leave nodes with longer links unconnected to the network due to an inability to send packets to it’s closest neighbour. The basestation where the data packets are collected is situated on a corner within the unit square. The area of the unit square and number of nodes in the network are varied to assess a range of network densities, sizes and scale. While the network is composed of homogeneous nodes the network conditions for each experiment will vary due to the noise profile in the network. The RPL routing protocol was used as the routing protocol for all experiments. RPL is a protocol that is currently under standardisation review. It is important to integrate any proposed standardised evaluation procedure within current standardisation efforts. RPL is claimed to have significant use cases within the BEMS application scenario and is well placed within the community to make a significant contribution to BEMS.

Three distinct solutions were implemented using RPL by using three different metrics to calculate routes: ETX, ETX-NH and ETT. This narrow set of solutions is chosen for a number of reasons. It demonstrates the ability of the response characterisation and modelling technique to distinguish between slightly differing solutions. Implementing a system that switches the communication context between many different whole communications solutions requires large amounts additional overhead as discussed in Section 6.3. A system that can make minimal changes to the network, such as switching between the metrics used, to provide a wider range of responses may prove more effective. RPL espouses the ability for a single routing protocol to elicit multiple responses from the network. It does this primarily by using different metrics and constraints to build the routing DODAG. The advantages, as stated, lie in the fact that changing between metrics is a less intensive process than switching between protocol.

ETX [30] is an extensively tested metric which has been advised as the default metric within the currently advised standard. ETX is focused on reducing the number of retransmissions to route a packet to a given destination. ETX if focused on delivering high data reliability on each packet transferred in the network, ETX-NH introduces a modification to the ETX metric, adding a mechanism that increases the ability of a solution to absorb change in the routing structure. ETT is based on the round trip time and is solely focused on building a DODAG that is focussed on delivering the packet sin the quickest time frame. the three metrics provide a wide berth of responses within the protocol.

In the TOSSIM simulation neither Received Signal Strength Indication (RSSI) or Link Quality Indicator (LQI) is available. These metrics would usually be used to measure the channel quality between nodes. As TOSSIM uses noise traces applied to the nodes to dictate the quality of the channel it is still possible to examine the network under multiple channel conditions. to replicate a real dynamic channel environment noise traces from real experiments are applied to the nodes. Since a real estimate of channel quality is not available we make a single distinguishing factor between difficult communications conditions and more manageable communications conditions. These factors are determined by the noise trace applied to the channels in the networks. The traces are taken from a set of publicly available set of real noise traces taken from a network in the Meyer library at Stanford University [41]. The “Meyer light” trace represents minimal interference noise while the “Meyer heavy” trace represents a more difficult communication scenario. This represents “light” and “heavy” interference in the channel.

**Figure 10:**
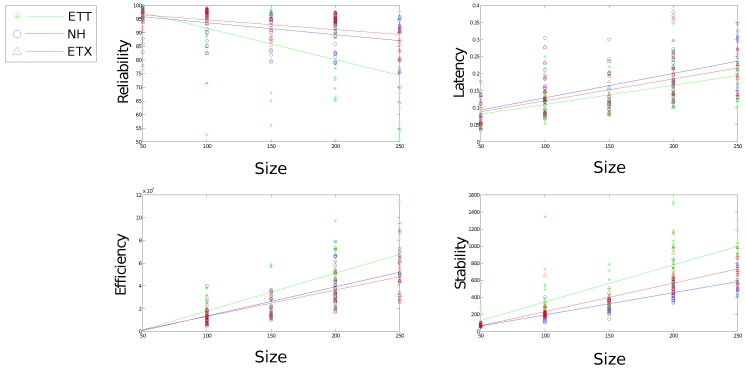
Network response from each solution as size varies.

**Figure 11:**
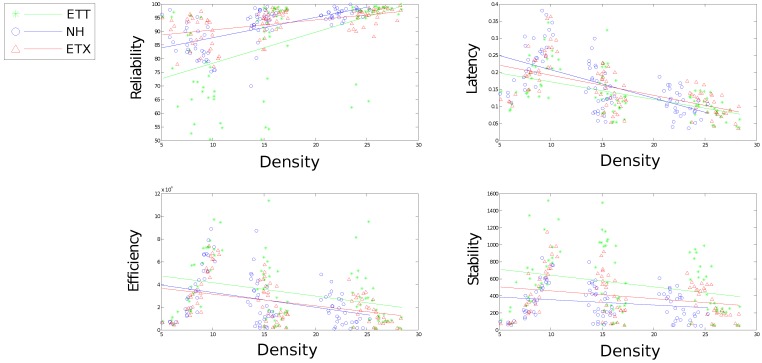
Network response from each solution as density varies.

**Figure 12:**
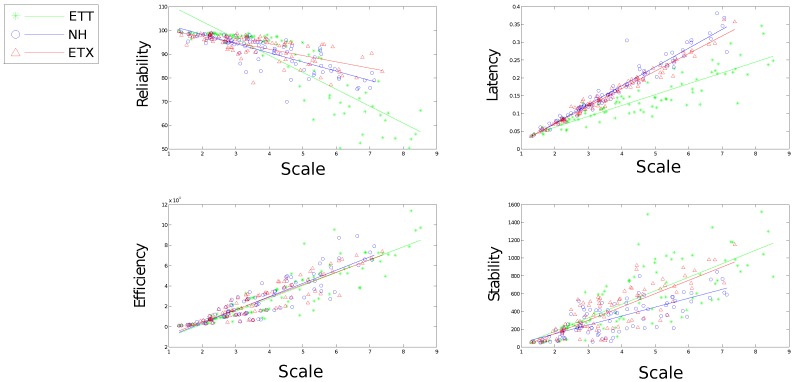
Network response from each solution as scale varies.

**Figure 13:**
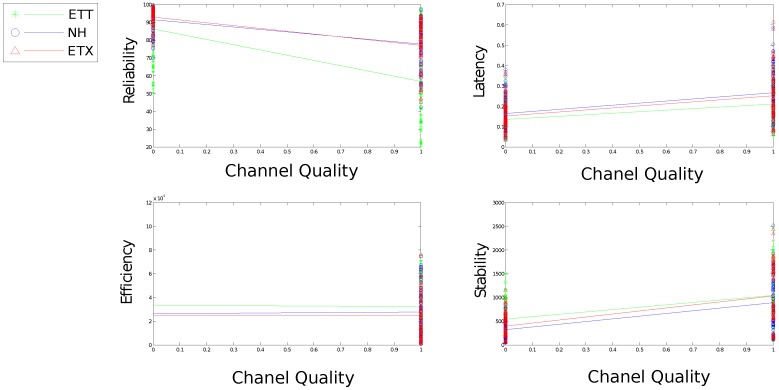
Network response from each solution as quality varies.

**Figure 14:**
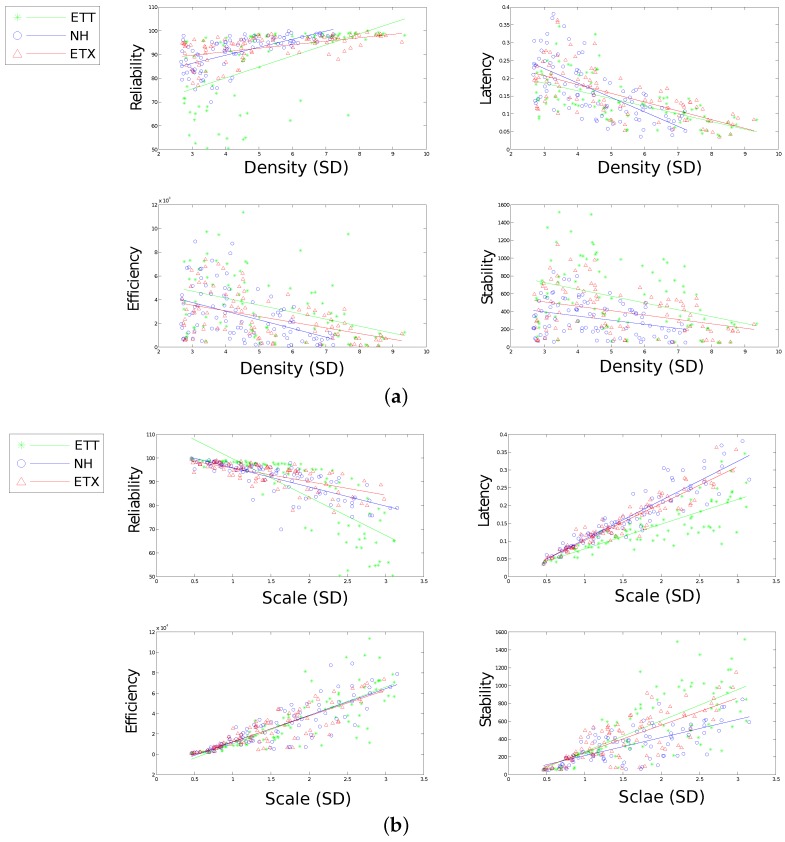
Results from simulation with regard to the secondary features. (**a**) Solution performance with regard to standard deviation of density; (**b**) Solution performance with regard to standard deviation of scale.

Figure 10, Figure 11, Figure 12, Figure 13 and Figure 14 display the statistics collected from the experiments. Figure 10, Figure 11 and Figure 12 show the network response for each solution as the density, scale and size of the network vary under the light trace. Figure 13 shows how the network response changes when the heavy trace is used. Data for the standard deviation of scale and density is shown in Figure 12.

Figure 10 shows all evaluation metrics following a general trend towards poorer results as size increases. Reliability and stability for both ETX and ETX-NH are well defined as size varies with latency and efficiency showing poor correlation.

Figure 11 shows a large impact on both reliability and latency effected by the change in density for all communication solutions. Neither efficiency nor stability are affected greatly by a change in density, however, density still proves an important tool for comparing each solution with regard to efficiency and stability. Each solution presents significant performance difference in terms of stability using density to compare solutions with NH presenting marked improvements over the other solutions. Both ETX-NH and ETX present an improvement over ETT with regard to energy efficiency.

High correlation between scale and each metric performance can be observed from Figure 12. This presents scale as a good indicator of how well a solution might perform in the network. It remains difficult to distinguish between solutions at small scales however higher contrast emerges as scale increases. A particular nuance with reliability is noticed for ETT over increasing scale. ETT shows improved reliability over both competitors for scale less than 4.5 hops. Over this threshold however ETT performs poorly in comparison. ETX and ETX-NH handles increasing scale more gracefully with regard to reliability.

Figure 13 presents each solutions performance under light and heavy channel interference. As expected, degraded channel quality has a significant effect on network reliability. Stability and latency are also heavily effected.

Figure 14 shows the performance of each of the solutions over a range of standard deviation of both density and scale in the network. The standard deviation are regarded as secondary features as they do not have as great an effect the performance as the primary features. The secondary features can however be used effectively for modelling and can be useful for characterising particular types of deployments which the primary features cannot distinguish. In these simulations the density (sd) can be used to distinguish between solutions with regard to stability and perhaps efficiency. Scale (sd) is effective at distinguishing between ETT and the other solutions with regard to latency.

It is clear that there are many insights to be garnered from the data presented. Certainly, the physical features chosen present a strong case for network performance characterisation visually. It is important that the performance can be modelled efficiently using the physical features for the purpose of solution comparison.

## 8. Discussion and Critique

The material presented in this article can be discussed in three distinct sections: the framework, the features and modelling technique used in evaluating the framework, and the dataset used for modelling and evaluation.

A major feature of the framework relies on an assumption that networks share a certain commonality. We assume that if we can find a set of characteristics that can well define the response in the network, this response will be reflected in another network that shares the same characteristics. This is an assumption that can be challenged and the idea of network commonality, or lack thereof, is addressed by Gnawali et al. [10] during testing over multiple testbeds. The observation made states that reliability can be heavily effected by a small number of fringe nodes that have tentative connection to the rest of the network. This effect can also have a large impact on network stability and can be highly specific to the deployment. The paper distinguishes reliability of nodes in the the 5th percentile to show this effect. This observation makes a case that a commonality between different networks may not be found. We believe, however, that the use of more physical metrics may accommodate for these unique traits within networks. For example, measuring the number of fringe nodes and their link quality may absorb this characteristic within the model.

The article uses a set of features including network size, scale, density and channel quality. This is not an exhaustive set of features with which to characterise a network. We believe that numerous other metrics, particularly with regard to the range of link qualities displayed within a network will be implemented and will offer useful insight and greater modelling accuracy. The physical features chosen in this article are a coarse means to characterise a network but which are those that have been used consistently in research articles to evaluate communications solutions. This can also be said for the modelling technique used in the article. Multiple linear regression modelling is a simple technique that provides a course but usable model for the purpose of evaluating the framework. It is important to note that the accuracy of any system built within the framework will be heavily dependent on the modelling technique but the framework itself is agnostic to the technique used.

One criticism that can be made of the dataset used in this article is that it is comprised solely on simulated data. While this is not ideal and cannot be considered comprehensive and fully conclusive testing it gives an initial indicator so that further investigation can be directed with promising results. As well as this the range of testing in terms of the physical characteristics must be increased. With IoT applications looking at larger scaled deployments these larger scales must be investigated in testing. As the simulation does not allow for LQI or RSSI measurements the channel quality is only measured over a binary scale; heavy or light interference. This issue can be resolved by introducing testbed and additional simulation experiments to the dataset. This can also be said for the homogeneity in the testing phase. Each node in the generated networks exhibits the same communication range. A noise trace is implemented on each network which ensures that communications conditions are not the same on each node however. We believe that heterogeneity with regard to the communications range is well captured in the physical features. Density and density (standard deviation) as well as any link measure would be heavily effected should large differences appear in the network with regard to communication range. One other metric that might help with the indication of range variability is the number of ‘leaf nodes’ in the network. Leaf nodes are nodes which collect and send data but never act as a relay in the network. This is not a physical feature but a product of how each solution creates the multihop network. Determining how well the physical features or additional features can detect or handle heterogeneity of communications range in the network should be investigated in any future work.

A final note on the framework is on the means of which the communications solution is deployed in the network. Initially we believe the framework will be used to direct the best choice of communications solutions to developers at the initial stages of a deployment. In order to achieve a fully autonomous system the issue of context switching during runtime must be meaningfully addressed. The purpose of Section 6.3 is not to address this question directly but to open discussion on possible mechanics to achieve this.

## 9. Conclusions and Future Work

This article introduces a framework which facilitates automatic and autonomous adaptation to application requirements within the network. This ensures the network is capable of delivering the best possible performance for the particular applications objectives. The framework uses different routing protocol and metric types as tools to create alternate communication solutions which elicit certain responses from the network. The framework then chooses the best solution to use given the network environment and the requirements stipulated by the application. The aim of which is to aid in wide-scale industrial IoT network deployment where choosing an appropriate solution for an application in a particular deployment might prove too expensive and time consuming.

To achieve this task each solution is evaluated and compared for their compliance to the applications preferences within a certain deployment. With automatic and autonomous behaviour essential, the framework effectively compares the solutions with minimal testing and without human exposure. Central to this concept is the idea that an IoT network can be modelled accurately to allow performance prediction. The article introduces a novel method of modelling the network using the physical features exhibited by a network and implements this with in the framework. Using this modelling technique the expected performance of a solution for each of the evaluation metrics can be determined. Developing pre-built models for use in the framework allows the assessment and comparison of solutions during network runtime. As the models are formed around physical features which are easily extracted from the network, a deployment assessment can be achieved autonomously and without excessive testing within a deployment.

The framework introduces a number of novel concepts for IoT communications:

- Taking advantage of the wealth of network tools already available to provide alternative performance characteristics while considering a macroscopic perspective on how each can facilitate the applications requirements in the network.
- Using pre-built network models to allow effective comparison between solutions during runtime.
- Proving the use of physical environment for IoT network modelling and determining a set of physical features from which network modelling is possible.

Using the modelling technique presented mixed results in terms of prediction accuracy are achieved when comparing the relative performance of evaluation metrics for each solution using a test set in simulation. The framework also presents a strategy for attaining the required features during runtime, matching and choosing the most appropriate solution as per the application requirements and deploying the solution for use in the network. The strength of the framework lies in a compartmental design where each facet can be replaced with an updated or alternative equivalent without compromising the overall framework.

The future direction for this work will be focused on introducing real testbed and production network data into the sample data set and refining the model to better characterise the different solutions. The simulations have presented some interesting results. The logical next step is to obtain a rich data set from real networks that display the range of physical features from which solution modelling can be achieved. From this extended data set a more refined model can be created. The framework is designed to be modular so that future performance models can be integrated easily. As well as this an extended rage of solutions will be tested using the same approach to build a set of solutions which are directly compared to each other. this will present a rough guideline for application developers when choosing a solution for a network deployment.

## Figures and Tables

**Table 1:** Pearson correlation between dependent variables.

Variable Pair	*ρ*	*p*-Value
Density-Scale	−0.5493	0.0009
Size-Density	8.9518 × 10^−4^	0.9832
Scale-Size	0.5843	0.0001

Source of data from experiments described in Section 7.

**Table 2:** The significant predictor variables for modelling the response of each evaluation metric as determined by a T-test.

Solution	Evaluation Metric	Predictor Variables					
		Density	Scale	Size	Density (sd)	Scale (sd)	Channel Quality
ETX-NH	Reliability	*	***				***
	Latency		***	**		*	***
	Efficiency		***	***	*		*
	Stability		*	**			***
ETX	Reliability	**	**	***			***
	Latency	**	***	**			***
	Efficiency		***	***	**	*	***
	Stability		**	***			***
ETT	Reliability		***	***		***	***
	Latency		**			**	***
	Efficiency		***	***		***	
	Stability		*	***			***

* *p*-value < 0.05; ** *p*-value < 0.01; *** *p*-value < 0.001.

**Table 3:** Model accuracy over a test data set.

		Reliability	Latency	Efficiency	Stability
1st	Percentage	76.68%	85.7%	50.43%	78.25%
	Precision	0.7257	0.6056	0.7267	0.8378
	Recall	0.6828	0.5860	0.6720	0.6667
	F1	0.7036	0.5956	0.6983	0.7425
2nd	Percentage	71.8%	61.92%	55.7%	63.25%
	Precision	0.6497	0.6228	0.4360	0.7569
	Recall	0.6183	0.5591	0.4032	0.5860
	F1	0.6336	0.5892	0.4190	0.6606
3rd	Percentage	81.39%	77.95%	59.81%	67.88%
	Precision	0.8107	0.8095	0.5152	0.7500
	Recall	0.7366	0.6398	0.4570	0.7258
	F1	0.7718	0.7147	0.4843	0.7377

**Table 4:** Physical attributes of the network.

Physical Feature	Value
Density	21
Scale	3
Size	137
Density (SD)	6
Scale (SD)	1
Channel Quality	light

**Table 5:** Scoring the criteria for each solution.

Solution	Criteria	Value	Criteria Score	Scaling Factor	Overall Score
ETX-NH	Reliability	96.29	2	1	2
	Latency	0.125	−12	N/A	−12
	Efficiency	19,570	7	0.5	3.5
	Stability	216.7	29	0.5	14.5
ETX	Reliability	96.44	2	1	2
	Latency	0.113	−2	N/A	−2
	Efficiency	18,520	13	0.5	6.5
	Stability	299.5	2	0.5	1
ETT	Reliability	90.86	−4	1	−4
	Latency	0.096	14	N/A	14
	Efficiency	25,520	−20	0.5	−10
	Stability	397.7	−31	0.5	−15.5

mean values: Reliability = $\frac{96.44+96.29+90.86}{3}$ = 94.53; Latency = $\frac{0.113+0.125+0.096}{3}$ = 0.111; Efficiency = $\frac{18520+19750+25520}{3}$ = 21,263; Stability = $\frac{299.52+216.73+397.69}{3}$ = 304.64.

**Table 6:** Solution scores after weighting for preference A.

		Preference A	
**Solution**	**Criteria**	**Weighting Criteria**	**Solution Score**
ETX-NH	Reliability	1 × 2 = 2	8 ✓
	Latency	1 × (−12) = −12	
	Efficiency	1 × 3.5 = 3.5	
	Stability	1 × 14.5 = 14.5	
ETX	Reliability	1 × 2 = 2	7.5
	Latency	1 × (−2) = −2	
	Efficiency	1 × 6.5 = 6.5	
	Stability	1 × 1 = 1	
ETT	Reliability	1 × (−4) = −4	−15.5
	Latency	1 × 14 = 14	
	Efficiency	1 ×(−10) = −10	
	Stability	1 × (−15.5) = −15.5	

✓ Winning solution.

**Table 7:** Solution scores after weighting for preference B.

		Preference B	
**Solution**	**Criteria**	**Weighting Criteria**	**Solution Score**
ETX-NH	Reliability	0.9 × 2 = 1.8	−3
	Latency	0.7 × (−12) = −8.4	
	Efficiency	0.2 × 3.5 = 0.7	
	Stability	0.2 × 14.5 = 2.9	
ETX	Reliability	0.9 × 2 = 1.8	1.9 ✓
	Latency	0.7 × (−2) = −1.4	
	Efficiency	0.2 × 6.5 = 1.3	
	Stability	0.2 × 1 = 0.2	
ETT	Reliability	0.9 × (−4) = −3.6	1.1
	Latency	0.7 × 14 = 9.8	
	Efficiency	0.2 × (−10) = −2	
	Stability	0.2 × (−15.5) = −3.1	

✓ Winning solution.

**Table 8:** Solution scores after weighting for preference C.

		Preference C	
**Solution**	**Criteria**	**Weighting Criteria**	**Solution Score**
ETX-NH	Reliability	0.6 × 2 = 1.2	−2.6
	Latency	0.8 × (−12) = −9.6	
	Efficiency	0.4 × 3.5 = 1.4	
	Stability	0.3 × 14.5 = 4.4	
ETX	Reliability	0.6 × 2 = 1.2	2.5 ✓
	Latency	0.8 × (−2) = −1.6	
	Efficiency	0.4 × 6.5 = 2.6	
	Stability	0.3 × 1 = 0.3	
ETT	Reliability	0.6 × (−4) = −2.4	0.1
	Latency	0.8 × 14 = 11.2	
	Efficiency	0.4 × (−10)= −4	
	Stability	0.3 × (−15.5) = −4.7	

✓ Winning solution.

**Table 9:** Three preference models used for weighting criteria.

	Pref. A	Pref. B	Pref. C
Reliability	100	90	60
Latency	100	70	80
Efficiency	100	20	40
Stability	100	20	30
